# Idiopathic humeral head osteonecrosis mimicking rotator cuff disorders: Two challenging diagnostic case reports

**DOI:** 10.1097/MD.0000000000018766

**Published:** 2020-01-17

**Authors:** Fang-Yu Kuo, Kuan-Lin Chen, Chieh-Chi Yen

**Affiliations:** a Department of Physical Medicine and Rehabilitation; b Department of Physical Medicine and Rehabilitation, Yumin Medical Corporation Yumin Hospital, Nantou; c Department of Radiology, Changhua Christian Hospital, Changhua, Taiwan.

**Keywords:** humeral head osteonecrosis, rotator cuff disorders, shoulder pain

## Abstract

**Rationale:**

Shoulder pain is a common complaint among patients, and rotator cuff disorders are the most common diagnoses. Humeral head osteonecrosis is easily masked by other more common diagnoses and concomitant conditions.

**Patient concerns:**

This challenging diagnostic report consists of 2 cases. Case 1 was that of a 59-year-old man who presented with right shoulder pain that had lasted for >1 year. Case 2 was that of a 52-year-old man who complained of right shoulder pain lasting for 6 months. They both presented with chronic right shoulder pain without relevant trauma history, and the physical examination showed a tenderness point over the right greater tuberosity.

**Diagnosis:**

These 2 patients were diagnosed with osteonecrosis involving the right greater tuberosity region via magnetic resonance imaging.

**Interventions:**

In case 1, the patient underwent cord decompression and artificial bone grafting with C-arm guidance. In case 2, the patient refused surgical intervention and decided to continue receiving physical therapy for symptom control.

**Outcomes:**

In case 1, the patient responded well to cord decompression and artificial bone grafting. After the surgery, the active range of motion was restored and the pain in the right shoulder diminished further. In case 2, conservative treatment helped alleviate the patient's shoulder pain but did not entirely eliminate it.

**Lessons:**

Physicians should always have a high index of suspicion for osteonecrosis, especially when treating chronic shoulder pain, regardless of whether there are typical symptoms/known risk factors or not.

## 1 Introduction

The humeral head is the second most commonly affected site by osteonecrosis (7% of all osteonecrosis patients), although it is considerably less commonly affected than the femoral head.^[1–3]^ Identifying humeral head osteonecrosis is deemed to be challenging, and it is difficult to suspect because its symptoms are initially nonspecific (pain and limited range of motion).^[4]^ The purpose of this report is to highlight the main features of humeral head osteonecrosis to promote early detection and prompt treatment for the improvement of outcomes. This report conforms to all CARE guidelines and reports the needed information accordingly.

## 2 Method

This is a report of 2 cases under the waiver of approval of the ethics committee or institutional review board. Written informed consents were obtained from these 2 patients for the publication of this report.

## 3 Case reports

### 3.1 Case 1

**Figure 1:**
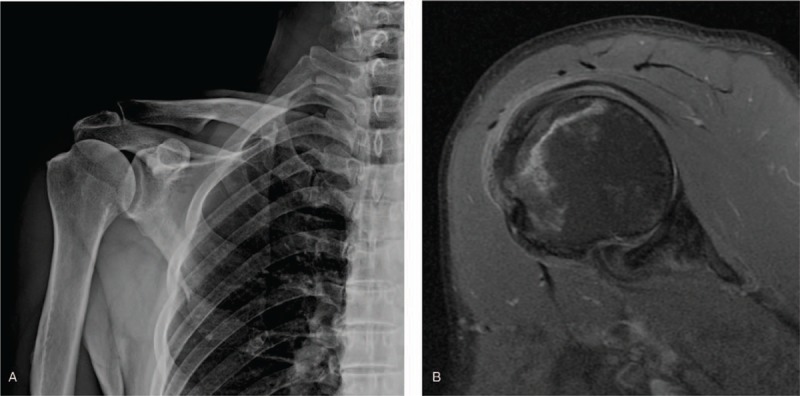
(A): A focal wedge-shaped defect on the superior lateral aspect of the right humeral head. (B): Right shoulder magnetic resonance imaging of the biceps axial view. The proton density fat-suppressed image shows a double-line sign with edema on the greater tuberosity region, a lesion compatible with osteonecrosis.

A 59-year-old man, without relevant trauma history, presented with right shoulder pain that had lasted for >1 year. He visited the Physical Medicine and Rehabilitation Outpatient Department for medical advice. Upon physical examination, a tenderness point over the right greater tuberosity of the humerus was found with limited range of motion. The plain radiograph showed a focal wedge-shaped defect on the superior lateral aspect of the right humeral head (Fig. 1A). The patient was subsequently diagnosed as having supraspinatus tendinosis and acromioclavicular joint arthropathy via musculoskeletal sonography.

The patient received physical therapy and hypertonic dextrose prolotherapy; however, some pain remained. A magnetic resonance image of the right shoulder revealed osteonecrosis of the greater tuberosity region and partial supraspinatus tendon tear (Fig. 1B). Investigation of conditions associated with osteonecrosis, such as corticosteroid use, infections, alcohol abuse, sickle-cell disease, and other systemic diseases, yielded negative results. The patient underwent cord decompression and artificial bone grafting with C-arm guidance in December 2018 under general anesthesia in the supine position. After the operation, the active range of motion was restored and the pain in the right shoulder diminished further. He had regular follow-up at the Physical Medicine and Rehabilitation Outpatient Department for >6 months. Follow-up magnetic resonance imaging 6 months after the surgery showed a normal right humeral head postoperative cord decompression appearance that is free of humeral head collapse.

### 3.2 Case 2

A 52-year-old man, without recent trauma history, originally visited the Neurology Outpatient Department due to numbness of his 4 limbs and tingling sensations on his feet. The neurologist ordered a nerve conduction study that revealed bilateral median neuropathies, left ulnar neuropathy, and probable lumbosacral radiculopathies. The patient also complained of right shoulder pain for the last 6 months with range of motion limitation; on physical examination, a tenderness point over the right greater tuberosity of the humerus that did not respond well to treatment with analgesics was found. Therefore, he was transferred to the Physical Medicine and Rehabilitation Outpatient Department for further evaluation.

**Figure 2:**
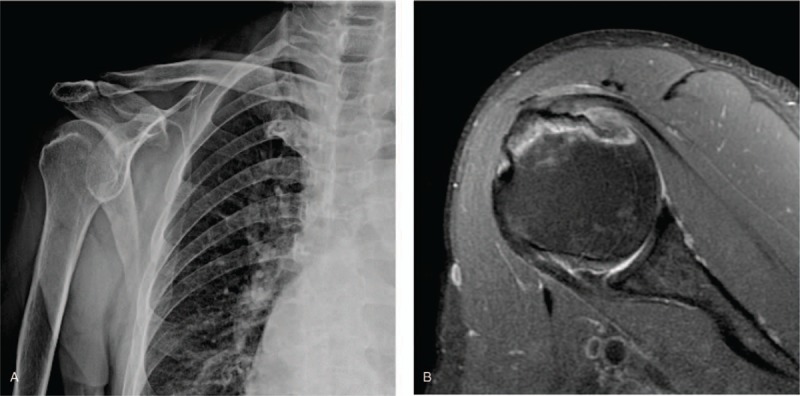
(A): A focal wedge-shaped sclerosis on the posterior aspect of the right greater tuberosity. (B): Right shoulder magnetic resonance imaging of the biceps axial view. Proton density fat-suppressed image shows a double-line sign involving the greater tuberosity, posterior and superior aspect of humeral head, a lesion compatible with osteonecrosis.

Right shoulder x-ray revealed a focal wedge-shaped sclerosis on the posterior aspect of the right greater tuberosity (Fig. 2A). Musculoskeletal sonography showed a defect over the infraspinatus insertion site on the greater tuberosity. Physical therapy including therapeutic ultrasound, interferential current therapy, and shortwave diathermy was arranged for the patient. However, 2 months later, the pain persisted. To confirm the diagnosis, we ordered a magnetic resonance imaging examination, which revealed osteonecrosis involving the right greater tuberosity, the posterior and superior aspects of the humeral head, and partial thickness tears of the supraspinatus and infraspinatus tendon (Fig. 2B). He refused surgical intervention and decided to continue receiving physical therapy for symptom control. Afterwards, he was regularly followed up at the Physical Medicine and Rehabilitation Outpatient Department for >6 months. Conservative management helped alleviate the patient's shoulder pain but did not entirely eliminate it. We also arranged image study follow-up for case 2, but he refused it despite being informed about the risk of disease progression. Therefore, we made further outpatient follow-up appointments to monitor his clinical condition.

## 4 Discussion

The humeral head is the second most commonly affected site by osteonecrosis. However, humeral head osteonecrosis accounts only for 7% of all osteonecrosis patients, which is considerably less common than femoral head osteonecrosis.^[1–3]^ This report aims to remind physicians to pay attention to the diagnosis of humeral head osteonecrosis through a comprehensive review of medical history and further examinations, so as to provide appropriate treatment for patients with chronic shoulder pain.

This report includes 2 cases of male patients, without relevant trauma history, who presented with right chronic shoulder pain and limited range of motion. Shoulder pain is a common complaint among patients during musculoskeletal consultation, with rotator cuff disorders being the most common.^[5]^ A primary care study on shoulder disorders found that rotator cuff tendinopathy accounts for 85% of patients with shoulder pain.^[6]^ Humeral head osteonecrosis is easily masked by other more common and concomitant diagnoses like rotator cuff disorders, adhesive capsulitis, and shoulder joint arthritis due to similar signs and symptoms. Shoulder pains that do not respond well to conservative treatment and become chronic require further examination (such as through magnetic resonance imaging) to allow the physicians to confirm the diagnosis and decide whether surgery should be indicated.^[7]^

Osteonecrosis had been accounted for the in situ death of a segment of bone.^[8]^ The etiologies and associated risk factors include mechanical vascular interruption, trauma, corticosteroid therapy, alcoholism, smoking, dysbarism (caisson disease), sickle-cell hemoglobinopathies, and other systemic diseases such as Gaucher disease and autoimmune diseases.^[1,4,8–10]^ Patients with humeral head osteonecrosis may present concomitant osteonecrosis of other joints, particularly the hip joint.^[11]^ Both of the patients reported having no relative predisposing conditions or risk factors for humeral head osteonecrosis. A careful review of medical history and physical examination can help physicians gain a better understanding of further examination and suitable treatment options for the patients. Even in the absence of relative predisposing conditions or risk factors, humeral head osteonecrosis should always be suspected in patients with chronic shoulder pain.

Studies suggested that typical humeral head osteonecrosis lesions are located on the medial aspect, in the glenohumeral contact site after ∼90° of shoulder abduction.^[12,13]^ These 2 cases initially presented with humeral greater tuberosity tenderness, where most rotator cuff tendons are inserted, and lacked the typical osteonecrosis site. The most widely used staging for humeral head osteonecrosis is the Cruess classification system for plain radiographic changes, modified from the Ficat–Arlet hip osteonecrosis classification. Stage I is not visible on the plain radiograph and can only be detected on magnetic resonance imaging. In stage II humeral head osteonecrosis, mottled sign and wedge-shaped sclerosis can be found. Stage III is characterized by the crescent sign, which represents subchondral fracture. Stage IV humeral head osteonecrosis is indicated by the presence of flattening of the humeral head with collapse, fragmentation, and loose bodies. Stage V is the terminal stage with degenerative changes involving the glenoid.^[2,14]^ The plain radiograph in case 1 showed a focal wedge-shaped defect on the superior lateral aspect of the right humeral head. In case 2, right shoulder x-ray revealed a focal wedge-shaped sclerosis on the posterior aspect of the right greater tuberosity. Further magnetic resonance imaging studies for these 2 patients resulted in a diagnosis of humeral head osteonecrosis on their greater tuberosity regions. According to the classification system of Cruess, both of the lesions in case 1 and case 2 were classified as stage II.

Once osteonecrosis is diagnosed, conservative management may be the treatment of choice for earlier stages.^[4]^ Non-steroidal anti-inflammatory agents and physical therapy may relieve painful range of motion.^[8,9]^ However, the patients must be informed about the risk of progression. As the disease progresses, advanced humeral head osteonecrosis and deteriorated humeral head collapse respond poorly after nonoperative management. Therefore, early detection and diagnosis are crucial to ensure that surgery is performed promptly. Various surgical methods have been used to successfully treat humeral head necrosis depending on the severity and staging of the disease, including cord decompression, partial resurfacing of the humeral head, and hemi-arthroplasty to total shoulder arthroplasty.^[9,15–19]^ In case 1, the patient underwent cord decompression and artificial bone grafting. The right shoulder joint's active range of motion was restored and the pain also diminished after the operation. He was regularly followed up at the Physical Medicine and Rehabilitation Outpatient Department for >6 months. Follow-up magnetic resonance imaging 6 months after the operation showed a normal postoperative appearance of cord decompression in the right humeral head without humeral head collapse. Case 2 was also classified as stage II humeral head osteonecrosis according to the Cruess classification system; however, the patient chose to continue receiving conservative treatment, such as physical therapy, and refused surgical intervention. The follow-up duration of case 2 was >6 months as well. Physical therapy alleviated the patient's shoulder pain but did not entirely eliminate it. We also arranged image study follow-up for case 2, but he refused it. Therefore, we arranged further outpatient follow-up appointments to monitor his clinical condition, and the patient was well informed of the risk of right humeral head osteonecrosis progression.

## 5 Conclusion

Physicians should always have a high index of suspicion for osteonecrosis, especially when treating chronic shoulder pain, regardless of whether there are typical symptoms/known risk factors or not. Moreover, early and accurate diagnosis may allow for proper treatment and improve patient satisfaction.

## Author contributions

**Conceptualization:** Fang-Yu Kuo, Kuan-Lin Chen.

**Supervision:** Kuan-Lin Chen, Chieh-Chi Yen.

**Writing – original draft:** Fang-Yu Kuo.

**Writing – review & editing:** Kuan-Lin Chen, Chieh-Chi Yen.

Fang-Yu Kuo orcid: 0000-0001-8469-2937.

Kuan-Lin Chen orcid: 0000-0003-0767-3219

Chieh-chi Yen orcid: 0000-0003-1693-4994
